# Electric Researches in Simian Poliomyelitis

**Published:** 1913-02

**Authors:** 


Electric Researches in Simian Poliomyelitis.—E. Bordet of Paris and V. Danulescu of Bucharest, *Arch. d'Elect. Med.*, October 25, 1912, refer to the initial studies of experimental poliomyelitis in monkeys by Landsteiner and Popper in 1909 and to the work of Levaditi, Flexner, etc. They detail various experiments, in the main showing an identical course in monkeys and in man, and conclude that the galvanic and faradic currents are harmless and of relative efficaciousness.

